# High SARS-CoV-2 Seroprevalence and Rapid Neutralizing Antibody Decline among Agricultural Workers in Rural Guatemala, June 2020–March 2021

**DOI:** 10.3390/vaccines10071160

**Published:** 2022-07-21

**Authors:** Chelsea Iwamoto, Kelsey E. Lesteberg, Molly M. Lamb, Diva M. Calvimontes, Kejun Guo, Bradley S. Barrett, Kaylee L. Mickens, Lindsey M. Duca, Jose Monzon, Anna N. Chard, Gerber Guzman, Edgar Barrios, Neudy Rojop, Kareen Arias, Melissa Gomez, Claudia Paiz, Guillermo Antonio Bolanos, Kathryn M. Edwards, Emily Zielinski Gutierrez, Eduardo Azziz-Baumgartner, Edwin J. Asturias, Mario L. Santiago, J. David Beckham, Daniel Olson

**Affiliations:** 1 Influenza Division, Centers for Disease Control and Prevention, 1600 Clifton Rd, Atlanta, GA 30329, USA; pgz5@cdc.gov (L.M.D.); mmn9@cdc.gov (A.N.C.); eha9@cdc.gov (E.A.-B.); 2 Division of Infectious Diseases, Department of Medicine, University of Colorado School of Medicine, 13001 E. 17th Pl, Aurora, CO 80045, USA; kelsey.lesteberg@cuanschutz.edu (K.E.L.); kejun.guo@cuanschutz.edu (K.G.); brad.barrett@cuanschutz.edu (B.S.B.); kaylee.mickens@cuanschutz.edu (K.L.M.); mario.santiago@cuanschutz.edu (M.L.S.); david.beckham@cuanschutz.edu (J.D.B.); 3 Department of Epidemiology and Center for Global Health, Colorado School of Public Health, 13199 E. Montview Blvd, Aurora, CO 80045, USA; molly.lamb@cuanschutz.edu (M.M.L.); edwin.asturias@childrenscolorado.org (E.J.A.); daniel.olson@childrenscolorado.org (D.O.); 4 Center for Human Development, Fundación para la Salud Integral de los Guatemaltecos, FSIG, Km 30 carretera de Coatepeque a Chiquirines Caballo Blanco, Retalhuleu 11010, Guatemala; mirella.calvimontes@fsigcu.org (D.M.C.); gerberguzman.estudioinfluenza@gmail.com (G.G.); ebarrios1908@gmail.com (E.B.); neudy.rojop.fsigcu@gmail.com (N.R.); kareen.arias@gmail.com (K.A.); melissa.gomez.fsigcu@gmail.com (M.G.); claudiapaiz27@gmail.com (C.P.); antonio.bolanos@cuanschutz.edu (G.A.B.); 5 La Comisión Presidencial de Atención a la Emergencia COVID-19 (Coprecovid), Guatemala City 01010, Guatemala; 6 Centers for Disease Control and Prevention, Division of Global Health Protection (CDC-DGHP), 1600 Clifton Rd., Atlanta, GA 30329, USA; rgi4@cdc.gov (J.M.); ebz0@cdc.gov (E.Z.G.); 7 Division of Infectious Disease, Department of Pediatrics, Vanderbilt University School of Medicine, 2200 Children’s Way, 6th Floor, Nashville, TN 37232, USA; kathryn.edwards@vanderbilt.edu; 8 Division of Infectious Disease, Department of Pediatrics, University of Colorado School of Medicine, 13123 E. 16th Ave., Aurora, CO 80045, USA

**Keywords:** COVID-19, SARS-CoV-2, seroprevalence, agricultural workers, cohort, Guatemala

## Abstract

Essential agricultural workers work under occupational conditions that may increase the risk of SARS-CoV-2 exposure and transmission. Data from an agricultural worker cohort in Guatemala, and anti-SARS-CoV-2 nucleocapsid IgG (anti-N IgG) testing were used to estimate past infections and analyze risk factors associated with seropositivity at enrollment and association with SARS-CoV-2 infection. The stability of neutralizing antibody (NAb) responses were assessed in a subset of participants. The adjusted relative risk (aRR) for seroprevalence at enrollment was estimated accounting for correlations within worksites. At enrollment, 616 (46.2%) of 1334 (93.2%) participants had anti-N IgG results indicating prior SARS-CoV-2 infection. A cough ≤ 10 days prior to enrollment (aRR = 1.28, 95% CI: 1.13–1.46) and working as a packer (aRR = 2.00, 95% CI: 1.67–2.38) or packing manager within the plants (aRR = 1.82, 95% CI: 1.36–2.43) were associated with increased risk of seropositivity. COVID-19 incidence density among seronegative workers was 2.3/100 Person-Years (P-Y), higher than seropositive workers (0.4/100 P-Y). Most workers with follow-up NAb testing (65/77, 84%) exhibited a 95% average decrease in NAb titers in <6 months. While participants seropositive at baseline were less likely to experience a symptomatic SARS-CoV-2 infection during follow-up, NAb titers rapidly waned, underscoring the need for multipronged COVID-19 prevention strategies in the workplace, including vaccination.

## 1. Introduction

Essential workers in the agricultural sector work under occupational conditions that may increase the risk of SARS-CoV-2 exposure and transmission. Workers often have limited access to sanitation facilities while harvesting crops and are in close proximity to one another inside packing plants and while using congregate transportation [1,2]. Lost wages because of missed work resulting from illness disincentivize symptom reporting and can result in workplace SARS-CoV-2 outbreaks. Compared with the general population, agricultural workers have a higher prevalence of chronic noncommunicable illnesses, such as chronic kidney disease of unknown origin, and may be at increased risk for severe disease resulting from SARS-CoV-2 infection [3,4]. In Guatemala, stringent public health measures, such as within-country travel restrictions, school closures, and curfews, were swiftly implemented following the first reported case of COVID-19, but essential agricultural activities were exempt from government restrictions [5]. Exempted from shelter-in-place mandates and unable to work remotely, many continued in-person work throughout the pandemic [1]. With increased vulnerabilities to SARS-CoV-2 infection and their critical role in global food security, agricultural workers represent an essential workforce that is also a disproportionately affected population.

While there is a growing body of literature using active COVID-19 case-surveillance and mortality data to estimate the burden of COVID-19 among agricultural workers [2,6,7], few serosurveys have reported on the risk of SARS-CoV-2 infections within this population [8,9]. Active surveillance with molecular testing is useful to identify acute COVID-19, but can underestimate the risk of SARS-CoV-2 infection and confound analyses of risk factors, if not coupled with serology [10]. Serologic SARS-CoV-2 antibody assays can identify previous mild COVID-19 and asymptomatic SARS-CoV-2 infections typically missed by traditional respiratory virus surveillance [11,12]. The addition of serology allows for more robust characterization of COVID-19 burden than routine case surveillance alone and provides an opportunity to better identify risk factors for SARS-CoV-2 infection among underreported groups used to inform workplace prevention interventions [13].

The immune response to SARS-CoV-2 infection is broad and involves complex inter-relationships between various immunological components including memory B and T cells and antibodies [14]. Neutralizing antibodies have been identified as an immune correlate of protection from symptomatic SARS-CoV-2 infection [15] and strongly correlate with anti-SARS-CoV-2 spike antibody titers [16,17]. Exploring the kinetics and stability of these immune components is important to understanding the durability of protective immunity against SARS-CoV-2 reinfection.

Previous analyses of pilot data from the “AGricultural workers and Respiratory Illness Impact (AGRI) Study” described an essential workforce highly vulnerable to the negative clinical and economic impacts of COVID-19 [18]. The study had yet to report accurate estimates of SARS-CoV-2 infection because seroprevalence findings were unavailable. Here, we determined seroprevalence among the cohort during the ‘first wave’ of the COVID-19 pandemic in Guatemala (June–December 2020) and the subsequent risk of COVID-19 by demographic and occupational characteristics. To explore neutralizing antibody (NAb) kinetics, we also assessed the stability of the NAb response in a subset of individuals who provided sera specimens twice during the surveillance period between June 2020 and October 2021.

## 2. Methods

The AGRI cohort was initially designed as an influenza cohort and was expanded to include SARS-CoV-2. The setting, population, and influenza-like illness (ILI) surveillance strategies have been previously described in detail [18]. Briefly, from June–December 2020, eligible workers at a large agribusiness—comprising nine worksites in the southwestern coastal lowlands of Guatemala—were offered enrollment in the study. Eligibility criteria included age >18 years, plans to continue employment with the agribusiness >1 year, access to a telephone, and agreement to allow use of company-based absenteeism and job performance records. At enrollment, participants provided baseline demographic, occupational, socioeconomic, and clinical data as well as a venous blood specimen for screening of anti-SARS-CoV-2 nucleocapsid (anti-N) IgG (Elecsys^®^ Anti-SARS-CoV-2 immunoassay, Roche Diagnostics) [19]. Active ILI surveillance strategies included self-reporting of workers’ symptoms to the study nurse during weekly worksite visits or anytime by telephone, daily ILI symptom screening of workers by supervisors, and sentinel surveillance of worker health posts located within the agribusiness. ILI was initially defined as fever >38 °C and cough in the last 10 days, but was expanded in January 2021 to include fever, cough or shortness of breath in the last 10 days to increase sensitivity of COVID-19 case detection [18]. Study nurses collected a nasopharyngeal (NP) swab from workers with ILI, and workers provided relevant clinical, epidemiologic, and socioeconomic data for themselves and their households. NP swabs were placed in viral transport media and tested within 24 h for SARS-CoV-2 utilizing the STANDARD Q COVID-19 rapid antigen test (STANDARD Q-NCOV-01G, SD *Biosensor*^®^, Inc., Suwon, Korea). Symptomatic participants unable to be seen by the study nurse for specimen collection received SARS-CoV-2 antigen testing through the Instituto Guatemalteco de Seguridad Social (IGSS). Results from SARS-CoV-2 testing performed by IGSS were verified by study nurses. Workers who tested positive for SARS-CoV-2 were required to quarantine at home for up to fourteen days and received 2/3 of base wages [18].

To explore NAb kinetics in relation to nucleocapsid IgG reactivities, a convenience sample of participants who were anti-SARS-CoV-2 nucleocapsid IgG positive at enrollment and had an additional blood specimen available 3–7 months post-enrollment were selected for additional antibody testing. Additional blood specimens for enrolled participants were obtained through an occupational health program, administered by the agribusiness. As a requirement of employment, workers regularly provide blood specimens for routine occupational health screenings. Enrolled participants provided informed consent for study access to banked blood specimens. Specimens from the convenience sample were then selected for NAb analyses based on representativeness of the age distribution of the cohort and antibody results consistent with one of three anti-N IgG reactivity patterns: 1. ‘N-decreasing’, where nucleocapsid IgG reactivities were higher in the 1st versus the 2nd time point; 2. ‘N-increasing’, where nucleocapsid IgG reactivities were lower in the 1st versus the 2nd time point, and 3. ‘N-vanishing’, where the 2nd specimen was seronegative.

## 3. SARS-CoV-2 Antibody Analysis

Total IgG antibody to SARS-CoV-2 nucleocapsid was determined using the Roche Diagnostics Elecsys anti-SARS-CoV-2 immunoassay as previously described [20]. All specimens were run in triplicate on the Roche Cobas e801 per the manufacturer’s instructions. The assay was previously reported to have a sensitivity of 95% and specificity of 99.8% at >14 days post-PCR positive SARS-CoV-2 patient specimens [20].

To evaluate the magnitude and stability of SARS-CoV-2 NAb responses, we utilized a lentivirus-based assay as previously described [21,22] (Figure S1A). Briefly, HIV-1Δenv with a nanoluciferase reporter (in kind gift from Dr. Paul Bieniasz, Rockefeller University) was pseudotyped with codon-optimized SARS-CoV-2 spike based on the ancestral Wuhan-1 strain in 293T cells [23]. Of note, this spike sequence is identical to the currently available vaccines. Pseudovirions (~3–4 × 10^5^ relative light units, RLU) were co-incubated with serial 5-fold dilutions of serum (from 1:10 to 1:31,250) in 100 µL complete media (F-12 Ham’s Medium with 10% FBS) containing 10,000 A549-ACE2 cells for 1 h at 37 °C [24]. The virus-serum-cell mixture was then plated into 96-well white plates, lysed after 48 h with Nano-Glo substrate (Promega) and RLUs were measured in a Victor X5 luminometer (Perkin Elmer). Fifty percent inhibitory concentrations (IC50) were calculated using a two-phase decay equation (GraphPad Prism). Figure S1B shows representative neutralization curves for seropositive specimens, including the World Health Organization international standard, 20/136, which exhibited a mean reciprocal IC50 titer of 2546 from 4 independent experiments [25]. We also evaluated 20 pre-COVID-19 pandemic specimens as negative controls. One specimen reached an IC50 of 17, but the remaining (95%) specimens tested had undetectable NAb titers (Figure S1C).

## 4. Statistical Analyses

To examine demographic and occupational characteristics associated with SARS-CoV-2 seropositivity at enrollment, we estimated relative risk (RR) using a modified Poisson regression with generalized estimating equations (GEE) to account for within worksite clustering. Worksites were included as a repeating variable with an exchangeable correlation structure. The modified Poisson regression model is a validated method to estimate relative risk for binary outcomes [26]. The model utilizes a log Poisson regression with robust variance estimation and may be combined with GEE to account for clustering [27]. This method was used because the log binomial multivariable regression model with GEE did not converge. We specified a priori to adjust for independent demographic, clinical, and occupational variables of interest (e.g., comorbid health conditions, number of household members, job type), in the multivariable model, as well as covariates shown in the bivariate models to be associated with seroprevalence at enrollment at a significance of 0.20. Statistical significance was assigned at a *p*-value < 0.05.

Incidence density (the number of cases/person-time of follow-up) of ILI and COVID-19-like illness CLI was calculated. CLI was defined using the original CDC case definition. To estimate the cumulative probability of SARS-CoV-2 infection by enrollment serostatus, we generated a Kaplan-Meier curve. As prior SARS-CoV-2 infection data was not available, enrollment date was established as day zero. All analyses were restricted to workers for whom baseline serology at enrollment was available and follow-up data was provided through 10 October 2021. Descriptive statistics, ILI and COVID-19 incidence densities, and regression analyses were conducted using SAS software (version 9.4, Cary, NC, USA).

To evaluate associations between sequential nucleocapsid IgG reactivities and kinetics of NAb responses, analysis of cut-off index (COI) values for SARS-CoV-2 IgG to nucleocapsid and SARS-CoV-2 NAb titer was completed using Prism software. Analysis of values was completed using two-way ANOVA with post-test analysis, t-test, and correlation analysis. A COI value >1 was considered positive for anti-nucleocapsid IgG, as specified by the manufacturer.

## 5. Ethical Oversight

The study was approved by the Colorado Multiple Institutional Review Board (COMIRB protocol #19-1836) and the Guatemala Ministry of Health National Ethics Committee (HRMC-560-2020). The Centers for Disease Control and Prevention relied on the Colorado Multiple Institutional Review Board (COMIRB). The local SW Trifinio Community Advisory Board for Research agreed to the study. Workers received no compensation for study participation. All study participants provided written informed consent to participate. Following a read description of the study by trained study nurses, participants were asked a series of questions to determine full understanding of the study and consent process. Information was reviewed as necessary. Once study nurses determined participants were fully informed, participants were permitted to sign the Consent Form. A copy of the Consent Form was provided to participants with the original maintained by the study.

## 6. Results

From June to December 2020, 1334 (93.2%) of enrolled workers provided serological specimens at enrollment. Workers with available baseline serology were representative of the overall cohort [18]; predominantly male (84%), young (mean age 31 years), and healthy (<13% self-reported underlying comorbid conditions) (Table 1). The most commonly reported chronic health conditions were obesity (9.2%) and kidney disease (3.5%). The majority of participants worked outdoors as field workers (71.0%) or managers (3.6%). All remaining participants held jobs within the plants as packers (23.8%), plant managers (1.0%), or in administration (0.7%). The largest percentage of seropositive participants were enrolled during October 2020 (*n* = 150, 24.4%) (Figure S2). Of the 1334 participants who provided blood specimens, 616 (46.2%) were anti-SARS-CoV-2 nucleocapsid IgG positive (Table 1). Of those participants working within the plants as packers (*n* = 317), 232 (73.2%) were seropositive at enrollment (Figure S3).

## 7. Risk Factors for Enrollment SARS-CoV-2 Seropositivity

Risk factors associated with seropositivity at enrollment are summarized in Table 2. In the unadjusted bivariate models, demographic characteristics associated with seroprevalence at enrollment included female sex (RR = 1.45, 95% CI: 1.26–1.66), indigenous ethnicity (RR = 1.23, 95% CI: 1.02–1.47) compared with Ladino ethnicity, number of household members (RR = 1.02, 95% CI: 1.01–1.03), and asthma (RR = 1.42, 95% CI: 1.02–1.96) (Table 2). Seropositivity at enrollment was also associated with liver disease (RR = 1.57, 95% CI: 1.22–2.02), cough within 10 days prior to enrollment (RR = 1.31, 95% CI: 1.08–1.59), and working as a packer (RR = 1.98, 95% CI: 1.64–2.39), or manager within the packing plants (RR = 1.86, 95% CI: 1.41–2.46) compared with those working in the field.

In the multivariable model, seropositivity was associated with reporting cough within 10 days prior to enrollment (aRR = 1.28, 95% CI:1.13–1.46), number of household members (RR = 1.01, 95% CI: 1.00–1.02) and job roles within the packing plant; workers in the packing plant (aRR = 2.00, 95% CI: 1.67–2.38) and plant managers (aRR = 1.82, 95% CI: 1.36–2.43) were approximately twice as likely to test seropositive at enrollment compared to counterparts who worked in the fields (Table 2).

## 8. Risk of COVID-19 by SARS-CoV-2 Nucleocapsid IgG Serostatus

**Figure 1:**
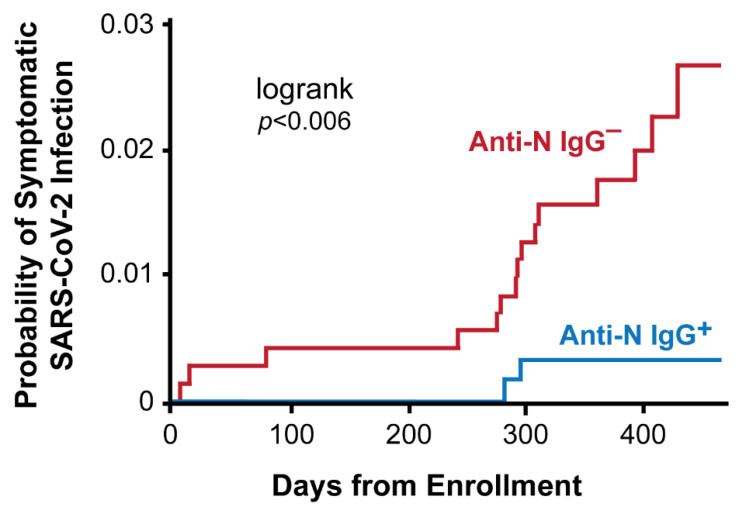
Cumulative probability of symptomatic SARS-CoV-2 infection by anti-SARS-CoV-2. nucleocapsid IgG serostatus at enrollment. Anti-N IgG + = anti-SARS-CoV-2 nucleocapsid IgG positive, indicates a prior SARS-CoV-2 infection detected through antibody testing at enrollment (*n* = 616). Anti-N IgG − = anti-SARS-CoV-2 nucleocapsid IgG negative, no evidence of a prior SARS-CoV-2 infection at enrollment (*n* = 718).

Between 15 June 2020, and 10 October 2021, the 718 participants who were anti-N IgG-negative at enrollment contributed 651.3 P-Y of follow-up and the 616 anti-N IgG-positive contributed 561.3 P-Y. Forty-six workers seronegative at enrollment (6.4%) developed ILIs (7.1/100 P-Y) during the follow-up period, of which 15 (32.6%) tested positive for SARS-CoV-2 (2.3/100 P-Y) a mean (SD) of 264.5 (131.6) days after enrollment. Twenty-five workers seropositive at enrollment (4.0%) developed ILIs (4.5/100 P-Y) during follow-up and 2 (8.0%) tested positive for SARS-CoV-2 (0.4/100 P-Y) at 281 and 294 days after enrollment, respectively. The probability of having a symptomatic SARS-CoV-2 infection during the follow-up period was lower among persons with versus without SARS-CoV-2 antibodies at baseline (log-rank *p* < 0.006) (Figure 1).

## 9. Stability of Neutralizing Antibody Responses among Nucleocapsid IgG Positive Individuals

Between June 2020 and March 2021, we identified 79 seropositive individuals with available baseline serology and a second serum specimen drawn during an occupational health visit, with an average time interval of 155 days (range: 95 to 191) between collection dates.

**Figure 2:**
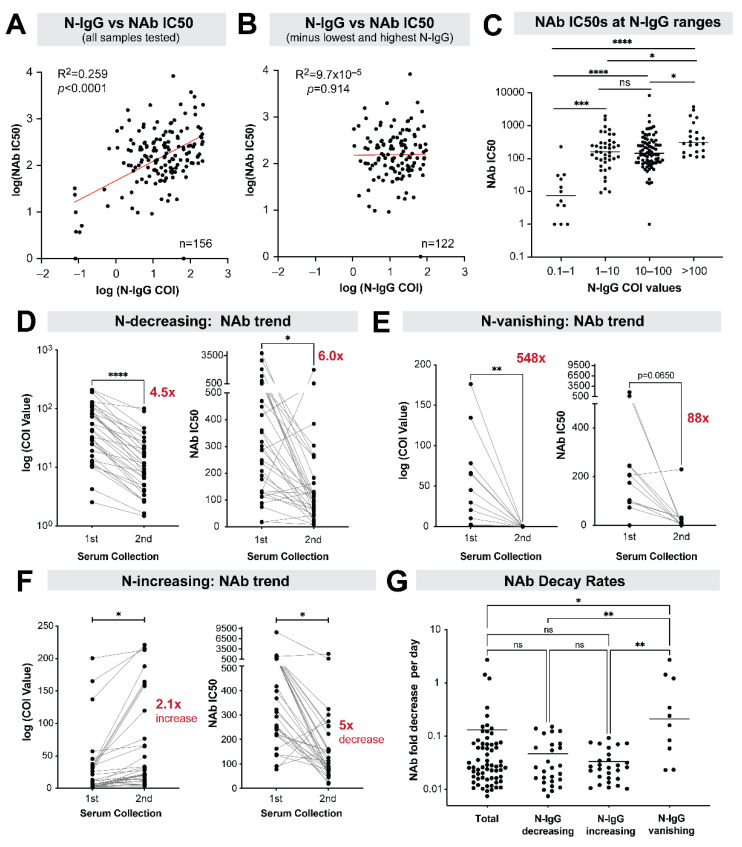
Assessment of neutralizing antibody responses among individuals with serum specimens. Collected at two time points and subjected to a lentivirus-based pseudovirion assay. (**A**) Correlations between NAb IC50s assessed against nucleocapsid IgG positive COI values using linear regression. (**B**) Serum specimens with COI values <1 and >100 removed. (**C**) Mean NAb IC50 values for 4 groups of nucleocapsid IgG COI values evaluated using ANOVA followed by Kruskal-Wallis test and grouped based on whether the nucleocapsid IgG positive COI values were (**D**) decreasing, (**E**) vanishing, or (**F**) increasing. Trends are highlighted in the graphs on the left of each panel. For each group, the NAb IC50 titers are assessed (right graphs on each panel) and differences evaluated using a 2-tailed paired Student’s t-test. (**G**) The fold-decrease in NAb divided by the time interval between serum collection compared between specimens with various nucleocapsid IgG reactivity trends using ANOVA and Kruskal-Wallis test. For all panels: * *p* < 0.05, ** *p* < 0.01, *** *p* < 0.001, **** *p* < 0.0001.

Two blood specimens, representing one from each of the paired specimens from two participants, were not of an adequate volume to perform NAb stability testing. Analysis of 156 serum specimens showed a substantial range in NAb IC50 values, with reciprocal NAb titers from undetectable to >2000. There was a significant correlation between nucleocapsid IgG titers (COI values) and the magnitude of NAb responses (Figure 2A). This correlation (R^2^ = 0.26, *p* < 0.0001) with NAb responses was lost when specimens with very low (COI = 0.1 to 1) and very high (COI > 100) nucleocapsid IgG titers were removed (Figure 2B). Very low and very high nucleocapsid IgG titers predicted low and high NAb responses, respectively, but could not distinguish intermediate titers (Figure 2C). The decrease or loss of IgG reactivity to SARS-CoV-2 nucleocapsid corresponded with a decrease in serum neutralizing capacity (Figure 2D,E), however, in participants with stable or increasing IgG reactivity to SARS-CoV-2 nucleocapsid, NAb titers continued to decrease over time (Figure 2F).

## 10. Subgroup Analysis of Antibody Neutralization Decay

The majority of individuals (*n* = 65, 84%) with serum specimens available (*n* = 77) for NAb testing at two time points had decreasing NAb titers (Table S1). When normalized to the time interval between blood specimen collection, we estimated a 0.13-fold decrease in NAb titer per day, translating to a 95% decrease in NAb titers within a 6-month period. NAb decay rates were significantly higher for nucleocapsid IgG-seropositive individuals who tested seronegative later (Figure 2G).

Upon evaluation of age-related differences, sex-differences, and presence of comorbid conditions, no specific risk factors were associated with decay of antibody neutralization over time.

## 11. Discussion

In a large cohort of Guatemalan agricultural workers, we found a high burden of COVID-19, with nearly half of the workers demonstrating evidence of prior SARS-CoV-2 infection by December 2020. We demonstrate that anti-nucleocapsid IgG positivity at enrollment was associated with a decrease in cumulative probability of subsequent COVID-19 over the subsequent year. In a subset of workers, however, anti-nucleocapsid IgG reactivity correlated with SARS-CoV-2 NAbs only at low or high titers and declined by 95% within 6 months. Within this essential workforce, seroprevalence at enrollment was substantial, especially among those who worked within the plant. Increased risk of SARS-CoV-2 infection was associated with job type; workers within packing plants demonstrated a 2-fold increase in risk of prior SARS-CoV-2 infection compared to those working in the fields, identifying a potential modifiable risk factor. These occupational risk factor findings can guide development of job-specific workplace interventions to decrease risk of SARS-CoV-2 infection.

Seroprevalence studies are useful in estimating the burden of COVID-19 as they help identify asymptomatic infections and subclinical disease typically missed by passive surveillance systems, which detect clinical illnesses [13,28,29,30]. Here, we show that approximately half of the 1334 workers tested positive for anti-SARS-CoV-2 nucleocapsid antibodies at study enrollment (June–December 2020). The high proportion of seropositivity is similar to SARS-CoV-2 burden estimates among agricultural workers in the U.S. and underscores the risks such essential workers take in maintaining food supplies during pandemics [8,9]. It also demonstrates that during a time in which non-pharmaceutical interventions were widely implemented across Guatemala, such as physical distancing measures, school closures, and masking, essential workers remained at risk of SARS-CoV-2 infection.

Increased risk of SARS-CoV-2 seropositivity at enrollment was associated with job roles within the packing plants compared to working in the fields. Cumulative incidence estimates of SARS-CoV-2 infection have varied across job roles in both the agricultural and food production sectors [2,9]. Drivers of workplace transmission are similar among these sectors and include the inability to physically distance, physically demanding work, poorly ventilated indoor spaces, insufficient or inadequate personal protective equipment, congregate living and transportation, and incentivized productivity standards that may encourage working while experiencing symptoms [31,32,33,34]. Transmission dynamics were not assessed in this analysis, but the association between seropositivity at enrollment and job roles suggests that workplace transmission may have contributed to the high seroprevalence estimates in this cohort.

While occupational prevalence of COVID-19 is not systematically reported, estimates of excess mortality attributable to the pandemic by occupational sector and occupation found that the food/agriculture sector in California had the highest relative mortality with an excess mortality of 39% compared to pre-pandemic periods [6]. A COVID-19 outbreak investigation between May and August 2020 among employees of a fruit grower in Okanogan County, Washington also found that relative risk for infection among employees packing and sorting fruit and those in other packing roles who worked primarily indoors in large groups were 2.7 and 2.4 times, respectively, than that of forklift operators who worked alone and partially outdoors [2]. Enhancing prevention education for workers with higher risk job roles, as well as managers who share responsibility in implementation of prevention measures, and workplace provision of personal protective equipment and vaccine may help to mitigate the risk of SARS-CoV-2 infection within this population.

Given that increased risk for SARS-CoV-2 infection is associated with occupational setting and job role, workplace prevention strategies are imperative to decrease COVID-19 incidence within the agricultural sector. Several workplace prevention measures implemented in agricultural and food processing settings have been suggested to mitigate worker risk for SARS-CoV-2 infection [1,32,33,34] and include onsite-testing and vaccination, use of plexiglass dividers, restricted building access for non-employees, increased frequency of worksite shuttles to minimize crowding, daily symptom checks and questionnaires, posted health education materials, close coordination with local public health authorities, and employer-provided personal protective equipment. In July 2020, the agribusiness instituted a systematic workplace prevention plan in accordance with guidelines set forth by the Ministerio de Salud y Asistencia Social [35]. Nevertheless, it is possible some workers were infected before these guidelines were fully implemented. Beginning in August 2021, agricultural workers were offered a no-cost SARS-CoV-2 mRNA vaccine through their employer. As of March 2022, approximately 99% of workers enrolled in the AGRI study had received two doses of the primary SARS-CoV-2 vaccine series. Vaccine acceptance has been shown to decrease when there is an associated out-of-pocket cost for vaccination [36], highlighting the importance of continued access to no-cost vaccine in the workplace to support continued SARS-CoV-2 mitigation through administration of booster doses. Annual serosurveys, including incorporation of anti-SARS-CoV-2-spike IgG testing to identify seroprevalence of vaccine induced antibodies and NAb testing, in combination with continued acute case surveillance of the cohort, will provide additional data as to the efficacy of these workplace mitigation strategies.

SARS-CoV-2 seropositivity among workers at enrollment was associated with a lower probability of having a symptomatic SARS-CoV-2 infection during the follow-up period, which may indicate some level of protection against reinfection in this cohort, though duration of protection has yet to be determined. SARS-CoV-2 infection mitigation measures, including vaccination, remain vital to worker health and livelihood [18], as unvaccinated individuals with a history of prior infection are twice as likely to become reinfected compared to vaccinated individuals with prior SARS-CoV-2 infection [37]. Among those previously infected with SARS-CoV-2, NAb activity was three times higher 2–3 weeks after two doses of mRNA vaccine compared with NAb activity 4–5 weeks post infection, [38] suggesting enhanced protection against re-infection. Additionally, the high transmissibility of the most recent SARS-CoV-2 variants underscore the importance of vaccination and treatment access in preventing severe disease outcomes; during the Omicron period, vaccine effectiveness for three doses of mRNA vaccine were found to provide similar effectiveness to prevent hospitalization as two doses of mRNA vaccine during the Alpha and Delta periods [39].

The availability of follow-up blood specimens from a subset of seropositive individuals allowed for assessments of the stability of anti-N IgG reactivity and neutralizing antibody responses. For the majority (85%) of participants with blood specimens from two timepoints, the anti-N IgG reactivities remained detectable within this timeframe of ~150 days. Nevertheless, the majority (84%) had a substantial decrease (>95%) in neutralizing antibody titers; this was most pronounced among individuals who were anti-N IgG positive at enrollment but were negative at the second time point. Although a decrease in anti-N IgG reactivity corresponded with decrease in serum neutralizing capacity, the continued NAb titer decay in participants with stable or increasing anti-N IgG reactivity suggests that nucleocapsid IgG titers do not provide a reliable surrogate for changes in antibody neutralization over time. Demographic or clinical risk factors were not associated with decay of antibody neutralization over time. The rapid decline in SARS-CoV-2 neutralizing antibody titers have been described in multiple COVID-19 cohorts, particularly among asymptomatic or mild cases [17,40,41]. The extent to which this rapid loss of humoral immunity would influence reinfections remain unknown and subject of on-going study in this cohort. Despite increased SARS-CoV-2 infections due to the Delta variant in Guatemala in the summer of 2021, as of October 2021, only two reinfections with clinical illness have been reported among workers (*n* = 616, 0.4/100 P-Y) who were anti-N IgG seropositive at enrollment. In addition to continued surveillance of humoral immunity against evolving SARS-CoV-2 variants, the role of T cell responses in reducing severe illness may become more important as the pandemic continues, given that T cell responses may be more stable and less prone to virus escape [14]. Advances in the rapid analysis of virus-specific CD8+ and CD4+ T cell responses from whole blood may facilitate these follow-up studies in the AGRI cohort [42].

The AGRI cohort design presents inherent strengths and weaknesses. Active ILI surveillance captures symptomatic SARS-CoV-2 infections more accurately than traditional passive surveillance mechanisms. Workers testing positive for SARS-CoV-2 were required to quarantine at home for up to two weeks and received reduced wages, likely suppressing reported symptoms during ILI surveillance. Nevertheless, this policy would not affect the validity of baseline serology to assess prevalence of infection among the cohort [18]. Originally designed as an influenza cohort and subsequently adapted for SARS-CoV-2 case identification, SARS-CoV-2 antigen testing was performed on workers reporting ILI symptoms (cough, fever, or dyspnea) and may underestimate COVID-19 incidence because of under-ascertainment of mild or subclinical infections [43,44]. Additionally, as serologic assay sensitivity is dependent on assay type, the severity of infection, and the timing at which the assay is administered relative to infection, it is possible that not all previously infected individuals in the cohort were captured through baseline serology [45,46]. Although our findings indicate that SARS-CoV-2 seropositivity at enrollment was associated with a lower probability of a symptomatic SARS-CoV-2 infection during follow-up, the analysis was not adequately powered to estimate associations between anti-N IgG titer levels and symptomatic COVID-19. Continued surveillance of the cohort may allow for future analysis of this association. While this paper focuses on workplace transmission, other factors associated with worksite (i.e., congregate meals and transportation) could not be adjusted for in our estimates.

## 12. Conclusions

High prevalence of anti-SARS-CoV-2 nucleocapsid antibodies was associated with occupational risk factors in this cohort, and seropositivity varied across job roles. Seropositivity at enrollment significantly reduced the cumulative probability of experiencing a symptomatic SARS-CoV-2 infection during follow-up, but the rapid decay of neutralizing antibodies underscores the need for multipronged SARS-CoV-2 infection prevention strategies, including vaccination. As new variants continue to emerge, workplace SARS-CoV-2 prevention strategies are imperative to reduce COVID-19 incidence within occupational settings and the surrounding communities. Given the breadth of institutional knowledge, workplace managers are uniquely positioned to implement and adapt public health measures to local context. These measures may extend beyond on-site practices, such as increasing the number of employer-provided work shuttles to decrease crowding on congregate transportation. Continued surveillance of the AGRI cohort in relation to the emergence of variants of concern, such as Delta and Omicron, will further inform the effectiveness of immune responses elicited in mild SARS-CoV-2 infections and allow for the continued assessment of SARS-CoV-2 infection risk to optimize infection prevention strategies.

## Figures and Tables

**Table 1:** Sociodemographic, clinical, and economic characteristics of cohort participants with baseline SARS-CoV-2 serology, Guatemala (*n* = 1334).

Demographics	
Age in years (mean, SD)	31.2 (8.5)
Male sex, *n* (%)	1125.0 (84.3)
Ethnicity, *n* (%): Ladino	581.0 (43.6)
Indigenous	92.0 (6.9)
Other *	3.0 (0.2)
Don’t know	658.0 (49.3)
**Clinical Data**	
Obesity (measured BMI> 30 kg/m^2^), *n* = 64	
Class 1 (BMI 30–<35 kg/m^2^)	54.0 (84.4)
Class 2 (BMI 35–<40 kg/m^2^)	9.0 (14.1)
Class 3 (BMI ≥ 40 kg/m^2^)	1.0 (1.5)
Kidney disease, *n* (%)	46.0 (3.5)
Blood disorder (e.g., anemia), *n* (%)	21.0 (1.6)
Cardiovascular disease (e.g., heart failure, CAD), *n* (%)	19.0 (1.4)
Diabetes, *n* (%)	13.0 (1.0)
Liver disease, *n* (%)	15.0 (1.1)
Asthma, *n* (%)	8.0 (0.6)
Pulmonary disease (e.g., COPD), *n* (%)	6.0 (0.5)
Neurologic disease (e.g., stroke), *n* (%)	8.0 (0.6)
Taking medications (Rx or OTC), *n* (%)	160.0 (12.0)
Ever received influenza vaccine, *n* (%)	75.0 (5.6)
ILI symptoms at enrollment, *n* (%)	33.0 (2.5)
Cough	23.0 (1.7)
Fever	13.0 (1.0)
SARS-CoV-2 IgG positive at enrollment, *n* (%)	616.0 (46.2)
**Work Conditions**	
Type of work, *n* (%)	
Administration	9.0 (0.7)
Field worker	947.0 (71.0)
Field manager	48.0 (3.6)
Packer (plant worker)	317.0 (23.8)
Plant manager	13.0 (1.0)
Individual monthly income, $USD, median (IQR)	337.7 (311.7, 376.6)
**Household Conditions**	
# of household members, median (IQR)	5.0 (4, 7)
# adults in household, median (IQR)	3 (2,4)
# children in household, median (IQR)	2 (1,3)
Household monthly income, $USD, median (IQR)	363.6 (311.7, 454.5)

BMI = body mass index, CAD = coronary artery disease, COPD = chronic obstructive pulmonary disorder, ILI = influenza-like illness, IQR = interquartile range Rx = prescription, OTC = over the counter * Other includes persons who identified as Garifuna or Trigueno.

**Table 2:** Unadjusted and adjusted * associations between risk factors and SARS-CoV-2 seroprevalence at enrollment among enrolled cohort participants with baseline serology, Guatemala (*n* = 1334).

Risk Factor	Unadjusted Relative Risk (95% CI)	*p*-Value	Adjusted Relative Risk (95% CI) *	*p*-Value
Age (years)	1.00 (0.99–1.01)	0.39	1.00 (0.99–1.01)	0.69
Sex				
Male	Ref		Ref	
Female	1.45 (1.26–1.66)	<0.0001	0.95 (0.87–1.04)	0.27
Ethnicity				
Ladino	Ref		Ref	
Indigenous	1.23 (1.02–1.47)	0.03	1.12 (0.99–1.26)	0.06
Unknown	1.08 (0.98–1.19)	0.13	1.06 (0.98–1.15)	0.17
Number of household members	1.02 (1.01–1.03)	<0.0001	1.01 (1.00–1.02)	0.04
Number of adults in household	1.03 (1.00–1.06)	0.06	-	-
Number of children in household	1.02 (1.00–1.05)	0.08	-	-
At least 1 comorbid health condition	1.04 (0.90–1.20)	0.60	-	-
Kidney disease	0.90 (0.71–1.12)	0.34	-	-
Blood disorder (e.g., anemia)	0.99 (0.68–1.44)	0.96	-	-
Cardiovascular disease (e.g., heart failure, CAD)	1.30 (0.97–1.75)	0.08	1.13 (0.87–1.47)	0.37
Diabetes	0.68 (0.38–1.23)	0.21	-	-
Liver disease	1.57 (1.22–2.02)	<0.001	1.26 (0.91–1.75)	0.17
Asthma	1.42 (1.02–1.96)	0.04	1.22 (0.97–1.53)	0.10
Pulmonary disease (e.g., COPD)	1.11 (0.62–1.99)	0.73	-	-
Neurologic disease (e.g., stroke)	1.31 (0.81–2.11)	0.27	-	-
Obesity	1.18 (0.86–1.64)	0.31	-	-
ILI symptoms within 10 days of enrollment	1.11 (0.88–1.41)	0.37	-	-
Cough	1.31 (1.08–1.59)	<0.01	1.28 (1.13–1.46)	<0.001
Fever	0.84 (0.53–1.32)	0.44	-	-
Type of work				
Field worker	Ref			-
Administration	0.59 (0.22–1.57)	0.29	0.61 (0.22–1.65)	0.33
Field manager	0.88 (0.61–1.27)	0.50	0.88 (0.61–1.28)	0.51
Packer (plant worker)	1.98 (1.64–2.39)	<0.0001	2.00 (1.67–2.38)	<0.0001
Plant manager	1.86 (1.41–2.46)	<0.0001	1.82 (1.36–2.43)	<0.0001
Individual monthly income, $USD (per $100 USD)	1.02 (0.97–1.08)	0.47	-	-
Household monthly income, $USD (per $100 USD)	1.02 (0.98–1.05)	<0.36	-	-

* Adjusted for age, sex, ethnicity, total number of household members, cardiovascular disease, liver disease, cough at enrollment, and type of work. CI = 95% confidence interval, CAD = coronary artery disease, COPD = chronic obstructive pulmonary disorder, ILI = influenza-like illness.

## Data Availability

The data presented in this study are available on request from the corresponding author. Restrictions apply to the availability of some of the data and therefore it has not been made publicly available.

## Supplementary Materials

The following are available online at https://www.mdpi.com/article/10.3390/vaccines10071160/s1, Table S1: Neutralizing antibody decay associated with sex, age, and comorbid health conditions, Figure S1: Lentivirus-based assay (A) and neutralizing antibody inhibition curves (B and C) utilized to evaluate the magnitude and stability of SARS-CoV-2 neutralizing antibody response, Figure S2: Participant anti-SARS-CoV-2 nucleocapsid IgG serostatus at enrollment and total number of participants with subsequent SARS-CoV-2 infection by enrollment month, Figure S3: Percentage of participants seropositive at enrollment by worksite and job type.
