# Supplementary material for: High SARS-CoV-2 Seroprevalence and Rapid Neutralizing Antibody Decline among Agricultural Workers in Rural Guatemala, June 2020–March 2021
Source: Vaccines (Basel). 2022 Jul 21;10(7):1160. doi: 10.3390/vaccines10071160 (PMC9323551; doi:10.3390/vaccines10071160)
Supplement: Supplementary file 1 [file vaccines-10-01160-s001.zip › Figure S1_revised_order_only.pdf]

Supplemental Figure S1. Lentivirus-based assay (A) and neutralizing antibody inhibition curves (B and C) utilized to evaluate the magnitude and stability of SARS-CoV-2 neutralizing antibody response

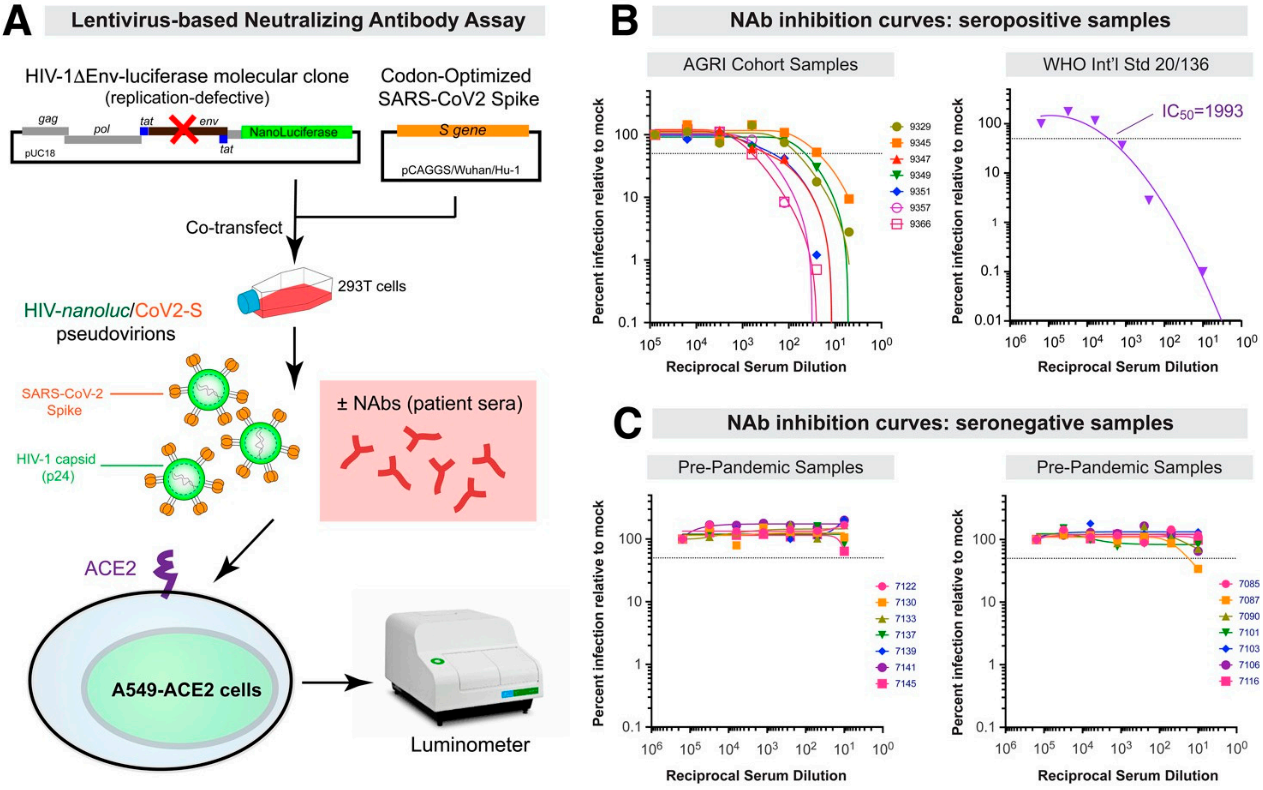

(A) the generation of HIV-1/nanoluciferase particles pseudotyped with the Spike gene of the Wuhan/Hu-1 strain. Pseudotype entry was assessed in A549-ACE2 cells. Typical neutralization curves for (B) seropositive AGRI cohort specimens and (C) an international serum standard. Dotted lines mark the serum concentration that results in 50% inhibition relative to mock.
