# Supplementary material for: High SARS-CoV-2 Seroprevalence and Rapid Neutralizing Antibody Decline among Agricultural Workers in Rural Guatemala, June 2020–March 2021
Source: Vaccines (Basel). 2022 Jul 21;10(7):1160. doi: 10.3390/vaccines10071160 (PMC9323551; doi:10.3390/vaccines10071160)
Supplement: Supplementary file 1 [file vaccines-10-01160-s001.zip › Figure S2_revised_order_only.pdf]

Supplemental Figure S2. Participant anti-SARS-CoV-2 nucleocapsid IgG serostatus at enrollment and total number of participants with a subsequent SARS-CoV-2 infection by enrollment month

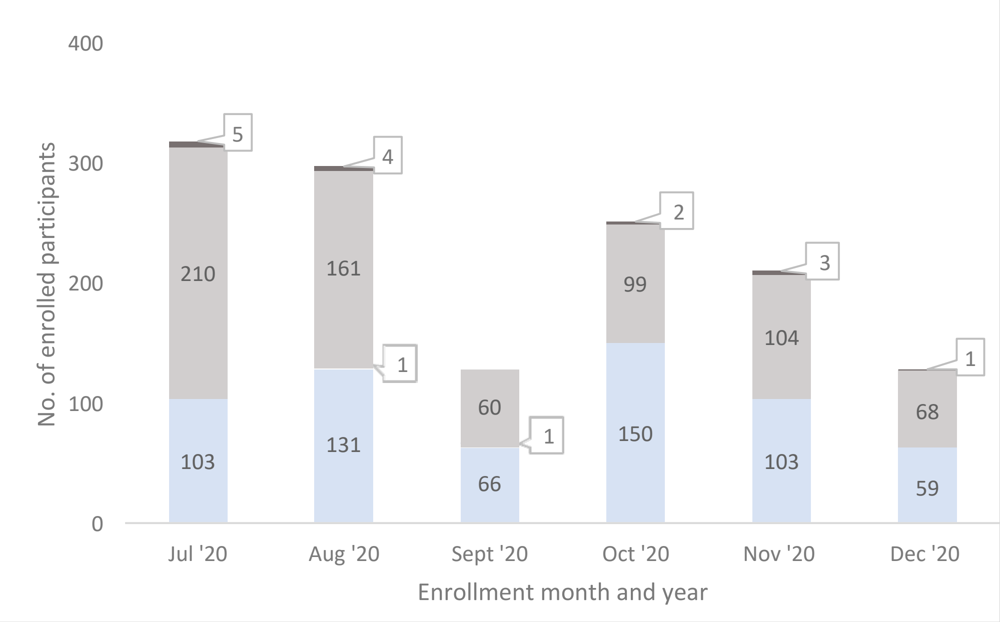

Light grey: SARS-CoV-2 seronegative participants at enrollment, dark grey: SARS-CoV-2 seronegative participants at enrollment with a subsequent SARS-CoV-2 infection during the follow-up period, light blue: SARS-CoV-2 seropositive participants at enrollment, dark blue: SARS-CoV-2 seropositive participants at enrollment with a subsequent SARS-CoV-2 infection during follow-up
