# Supplementary material for: High SARS-CoV-2 Seroprevalence and Rapid Neutralizing Antibody Decline among Agricultural Workers in Rural Guatemala, June 2020–March 2021
Source: Vaccines (Basel). 2022 Jul 21;10(7):1160. doi: 10.3390/vaccines10071160 (PMC9323551; doi:10.3390/vaccines10071160)
Supplement: Supplementary file 1 [file vaccines-10-01160-s001.zip › Figure S3_revised.pdf]

Supplemental Figure S3. Percentage of participants seropositive at enrollment by worksite and job type

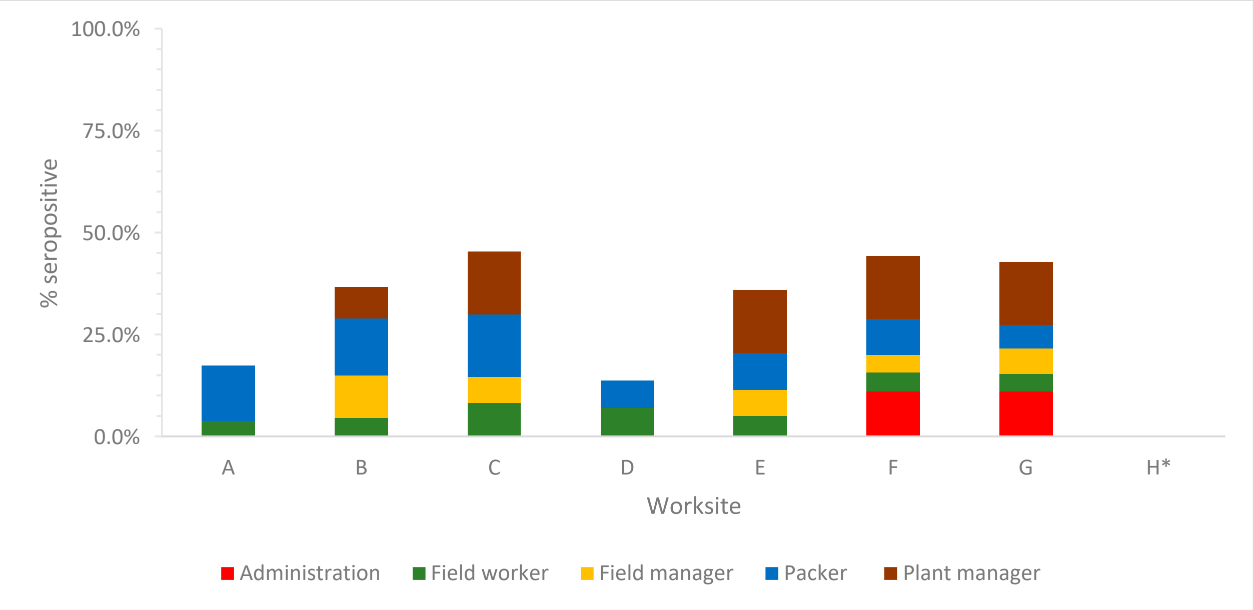

Worksite "I" is omitted as no participants reporting to this site were seropositive for SARS-CoV-2 at enrollment
*One field worker of n=3 participants reporting to worksite "H" was seropositive at enrollment
