# Supplementary material for: High SARS-CoV-2 Seroprevalence and Rapid Neutralizing Antibody Decline among Agricultural Workers in Rural Guatemala, June 2020–March 2021
Source: Vaccines (Basel). 2022 Jul 21;10(7):1160. doi: 10.3390/vaccines10071160 (PMC9323551; doi:10.3390/vaccines10071160)
Supplement: Supplementary file 1 [file vaccines-10-01160-s001.zip › Table S1_revised.pdf]

Supplemental Table S1. Neutralizing antibody decay associated with sex, age, and comorbid health conditions

	All participants with decreasing NAb titers (N = 65)		Decreasing NAb titers with outliers removed (N = 62)*		Participants with decreasing anti-nucleocapsid IgG COIs (N = 47)	
	**Slope of Ab decay**	**P-value**	**Slope of Ab decay**	**P-value**	**Slope of decrease**	**P-value**
**Age at enrollment**	0.009	0.15	-0.0007	0.47	-0.009	0.42
**Sex (female)**	-0.12	0.63	-0.03	0.37	-0.12	0.58
**Presence of comorbidities****	-0.11	0.57	-0.02	0.46	-0.19	0.49

NAb=neutralizing antibodies, COI=cut off index

*A subset (n=12) of the 77 participants with sera available for NAb testing at two timepoints were excluded due to NAb increases between the first and second collection dates.

**Comorbidities means reporting having at least one of the following conditions at enrollment: BMI > 30 kg/m^2, pulmonary disease, neurologic disease, liver disease, kidney disease, cardiovascular disease, diabetes, asthma, blood disorder; ~14% of workers in the sub-analysis had at least 1 comorbidity.
